# Correlates of Research Effort in Carnivores: Body Size, Range Size and Diet Matter

**DOI:** 10.1371/journal.pone.0093195

**Published:** 2014-04-02

**Authors:** Zoe M. Brooke, Jon Bielby, Kate Nambiar, Chris Carbone

**Affiliations:** 1 Institute of Zoology, Zoological Society of London, London, United Kingdom; 2 Brighton and Sussex Medical School, University of Sussex, Brighton, United Kingdom; Bangor University, United Kingdom

## Abstract

Given the budgetary restrictions on scientific research and the increasing need to better inform conservation actions, it is important to identify the patterns and causes of biases in research effort. We combine bibliometric information from a literature review of almost 16,500 peer-reviewed publications on a well-known group of 286 species, the Order Carnivora, with global datasets on species' life history and ecological traits to explore patterns in research effort. Our study explores how species' characteristics influenced the degree to which they were studied (measured as the number of publications). We identified a wide variation in intensity of research effort at both Family and Species levels, with some of the least studied being those which may need protection in future. Our findings hint at the complex role of human perspectives in setting research agendas. We found that better-studied species tended to be large-bodied and have a large geographic range whilst omnivory had a negative relationship with research effort. IUCN threat status did not exhibit a strong relationship with research effort which suggests that the conservation needs of individual species are not major drivers of research interest. This work is the first to use a combination of bibliometric analysis and biological data to quantify and interpret gaps in research knowledge across an entire Order. Our results could be combined with other resources, such as Biodiversity Action Plans, to prioritise and co-ordinate future research effort, whilst our methods can be applied across many scientific disciplines to describe knowledge gaps.

## Introduction

Biodiversity is declining at an unprecedented rate [1] and the reduction of future losses depends, at least in part, on a comprehensive and balanced understanding of the natural world. It is well known that gaps and biases exist in knowledge of the natural world [2]–[5] but not the extent, reasons or mechanisms behind them. There is, however, an increasing awareness of the need to identify and quantify these gaps, particularly in ecology and conservation [3]–[9] where the mismatch between science and conservation action is well-documented [11]–[16]. Certain clades and their constituent species are better-studied than others [2]–[5], [7], [17], but there is little understanding of which ecological and life history characteristics are associated with being well-studied.

Ecological and conservation science is likely to be driven by a similar complex web of factors that drives scientific research in general [16]. There is an entire discipline, “science technology studies”, which focuses on how these factors shape the biases inherent in science. These factors range from the individual scientist to much broader socio-political, cultural and economic values.

Biases in research effort may be influenced by practical considerations such as ease of study which may be related to species abundance, larger geographic ranges, whether species are found in convenient-to-study locations or the complexities of studying ecosystems [11]–[16], [18]–[21]. Indeed, this is the most frequently cited contributory factor to the conservation science-action mismatch [11]–[16]. Academic scientists may also be influenced by the use of performance indicators which encourage publication in high impact journals with much less recognition for undertaking practical conservation work or on disseminating findings more widely and accessibly, albeit with less easily quantified “impact” [10], [12]. It is too early to speculate whether these performance indicators will affect the collection and publication of simple natural history data which vitally underpin conservation action by assessing threats and clarifying priorities [10], [22].

Intensity of research effort may also be influenced by how “attractive” or “charismatic” species are to humans, for example there is some evidence to suggest that humans are more attracted to larger animals [23]–[28]. Researchers may also find purely predatory species more attractive, which may lead to a preference of studying clades according to their diets. Whilst the amount of research effort focused on different species will be related to their biological and geographical characteristics, these characteristics influence research interests in different and sometimes conflicting ways. Previous research has examined, at higher taxonomic levels, factors influencing which species are better-studied [2]–[5]. One trend is the consistently identified negative relationship between extinction risk and research effort (measured as number of published papers). In addition, there is a strong taxonomic bias in the literature, with an over-representation of birds and mammals [3], [5] to the detriment of less well-studied but more threatened taxa such as amphibians [3], [4], [8]. On a finer-scale, taxonomic bias exists even within well-studied clades, for example the Order Carnivora dominates the conservation literature within mammalian research [2].

Here we present a set of analyses on the research effort within the Order Carnivora (hereafter referred to as “Carnivores”) aimed at identifying which biological and ecological traits can predict the level of research attention a species receives. We chose Carnivores as our study Order because we needed a relatively intensively studied group in order to detect any differences in research effort and whether any of these differences were associated with species' traits. The Order contains a large number of near-globally distributed species which vary considerably in ecology and behaviour (such as group size, diet, geographic distribution) and life history traits (for example body mass, age at sexual maturity). Whilst the majority of Carnivores are terrestrial, there are 37 largely marine species (*Enhydra lutris*, sea otter; *Odobenus rosmarus*, walrus; 19 seals in Family Phocidae; 16 eared seals in Family Otariidae).

Exploring patterns of research effort in relation to species' traits may begin to reveal scientists' motivation for selecting and focusing on particular types of study species. By combining bibliometric information with global datasets on the ecology and life history of Carnivores, we can address the following specific questions with a view to elucidating patterns and trends in peer-reviewed literature, and determining the mechanisms underpinning these patterns:

1. What is the level of disparity in research effort among Carnivore Families and Species?

2. What intrinsic and extrinsic factors determine a Species' level of research effort?

## Methods

We obtained a list of 286 Carnivores in 15 Families based on Wilson and Reeder's [29] mammalian taxonomy from the PanTHERIA database [30]. We used bibliometric methods to quantify variation in species' research effort and collected peer-reviewed papers from Thomson Reuters' “Web of Science” (WoS) database for all Carnivores. We used SQLite to create the final database by combining the bibliometric data with intrinsic and extrinsic species' characteristics from PanTHERIA [30]. This database was queried using the RSQLite package for R version 3.0.2 [31]. All further analyses were also conducted using R [31].

### Bibliometric data collection and extraction

Literature searches were carried out for each individual Species using their scientific binomial(s) and any synonyms. All papers published from 1900 to the end of 2010 were included. In an attempt to maximise the relevance of the papers retrieved whilst maintaining broad subject coverage, we used integral WoS search tools to limit papers to one (or more) of sixteen Science Citation Index biological categories such as “ecology” and “zoology” (see Material S1).

The filtered papers were downloaded from WoS and saved as text files. A parser written in R [31] was used to extract and tabulate relevant data from the downloaded files. We extracted data on the first author, publication year, title, journal title, abstract and number of times cited.

To ensure that the focal species (the subject of the search) was the subject of each included study, we manually checked the title of each paper in the database and removed those where this was not the case (for example biomedical papers using species as experimental subjects). Where the species of interest was not mentioned in the title, the abstract was checked. In many instances irrelevant papers passed through our initial filter because the name of the species of interest appeared in “Keywords Plus” which are index terms created by Thomson Reuters from frequently occurring words in the titles of an article's cited references. If the paper was not about the species of interest, it was removed from the database. Where more than one species was the focus, the paper was counted for each of the study species. Papers on free-ranging, feral populations of domestic dog (*Canis lupus familiaris*), domestic cat (*Felis catus*) and domestic ferret (*Mustela putorius furo*) were included but papers on these species as companion animals (pets) were excluded.

### Intrinsic and extrinsic factors determining research effort

We investigated how a number of potential factors affected research effort by collating data on life history and ecological traits. Data on these traits were obtained from the PanTHERIA database [30], a comprehensive dataset containing information on all known extant and recently extinct species. All traits were defined as outlined in [30]. We used the following traits in our analyses:

- Family based on Wilson and Reeder's [29] mammalian taxonomy from PanTHERIA [30].
- Adult body mass.
- Geographic range size based on the extent of occurrence (distribution).
- Diet breadth (number of dietary categories eaten) - a continuous variable ranging from a single dietary category (highly specialist) to seven (generalist omnivore). Specialists may be of greater research interest than generalists.
- Habitat breadth (number of habitat layers used) and activity cycle (nocturnal, diurnal or mixed cycle) - potential indicators of how accessible a species may be to researchers.
- Mean human population density across geographic range (persons per km^2^) – a summary measure of anthropogenic impact [32].
- Information on species conservation status was obtained from the IUCN Red List [33], the most widely used, best-suited metric of risk available [34].

Although the PanTHERIA database is extensive, data were not available on every trait for every species but all variables included in our analyses had a minimum of 70% coverage (see Figure S1).

### Analyses

Simple descriptive bibliometric analyses (such as number of papers per group) were performed to determine research effort among Carnivores by Family, Species and extinction risk. We created a subset of the 37 marine Carnivores and present simple descriptive analyses for this group compared with their terrestrial counterparts.

Although the IUCN Red List was first proposed in 1963, more rigorous assessment categories were introduced in 1994 so we created another subset comprised of all papers published from 1995–2010 to explore the possible effect of IUCN threat status on research effort. Kruskal-Wallis testing was used to identify whether there were significant differences between the numbers of papers published in each IUCN category. *Post-hoc* pair-wise Wilcoxon tests with Bonferroni correction were then used to identify which of the pair-wise comparisons were responsible for the overall difference detected by the Krusal-Wallis test.

Generalised Linear Modelling (GLM) was used to quantify the relationship between the number of publications and the intrinsic and extrinsic species factors. As the response variable was a count (number of publications), which included a number of ‘zero’ observations, the GLM was initially modelled using Poisson distribution which does not predict negative values and has a mean-variance relationship that allows for heterogeneity. However, diagnostic model scatter plots of the standardised residuals revealed considerable over-dispersion and the dispersion parameter (φ) was greater than 100 for all variables. Rather than transforming the response data which can result in poor model performance [35], we corrected the standard errors using a quasi-GLM (where variance is given by dispersion parameter φ multiplied by the mean μ) which resulted in a slight improvement. There were no patterns in the plotted residuals using a negative binomial model with logarithmic link. Consequently, negative binomial GLMs were carried out using the glm.nb function of the MASS package [36] and Standardized Beta coefficients were obtained using the lm.beta function of the QuantPsyc package [37] in R version 3.0.2. [31].

Eight potential predictor species variables were used: adult body mass, geographic range size (hereafter “range”), mean human population density (hereafter “HPD”), Family, IUCN status, diet breadth, habitat breadth and activity cycle. To meet the assumption of linearity in log-frequency space, adult body mass and HPD were log_10_ transformed. Range was square-root transformed. The eight potential predictor variables were incorporated into a full multivariate GLM. Minimal adequate model selection was based on the hypothesis-testing backwards-simplification approach using the *z*-statistic, removing the least significant term before refitting the model and used likelihood-ratio tests to compare the two models. Possible interactions between adult body mass, range and HPD and between adult body mass, range and IUCN status were included in the models. We examined the minimal adequate model residuals to identify any species with much more or much less research effort than our model would predict based on their biology. The marine Carnivores were excluded from this GLM due to absence of range and HPD data; instead we carried out separate univariate GLM to explore the relationship between the number of publications on marine Carnivores and adult body mass, diet and IUCN status.

Many biological predictor variables are known to intercorrelate: Pairwise correlations identified statistically significant relationships between body mass and range (r_τ_ = 0.2046, *p*<0.001) and between range and human density (r_τ_ = −0.1348, *p*<0.005). A frequently cited strategy to deal with multicollinearity is to retain only the predictor variable which is most strongly correlated with the response variable [38]. However, this strategy is only appropriate where the intercorrelated terms measure the same thing; dropping one or more highly correlated variables implicitly redefines the research question [39]. As there was no compelling evidence for removing one of the variables, they were all retained. Variance Inflation Factors (VIF) calculated for each set of variables in the multivariate model (Tables S1A and S1B) were low (≤2.5) suggesting that collinearity was unlikely to significantly affect it.

The temporal trend in annual proportion of publications concerning at-risk species (IUCN Near Threatened, Vulnerable, Endangered and Critically Endangered) compared to non-threatened species was assessed using a Binomial GLM. Temporal autocorrelation was found in the model residuals hence a Newey-West [40] covariance estimator with a lag of 1 was used to derive robust standard errors (Sandwich package [41], [42] for R version 3.0.2 [31]). Only the last 50 years of data were modeled (1960–2010) due to the extremely low number of annual publications prior to this date.

## Results

During the data collection period a total of 23,655 papers were downloaded, of which 16,367 were included in the final dataset. The median number of papers per species was 10 (mean 57; range 0–923).

### Temporal trends

**Figure 1:**
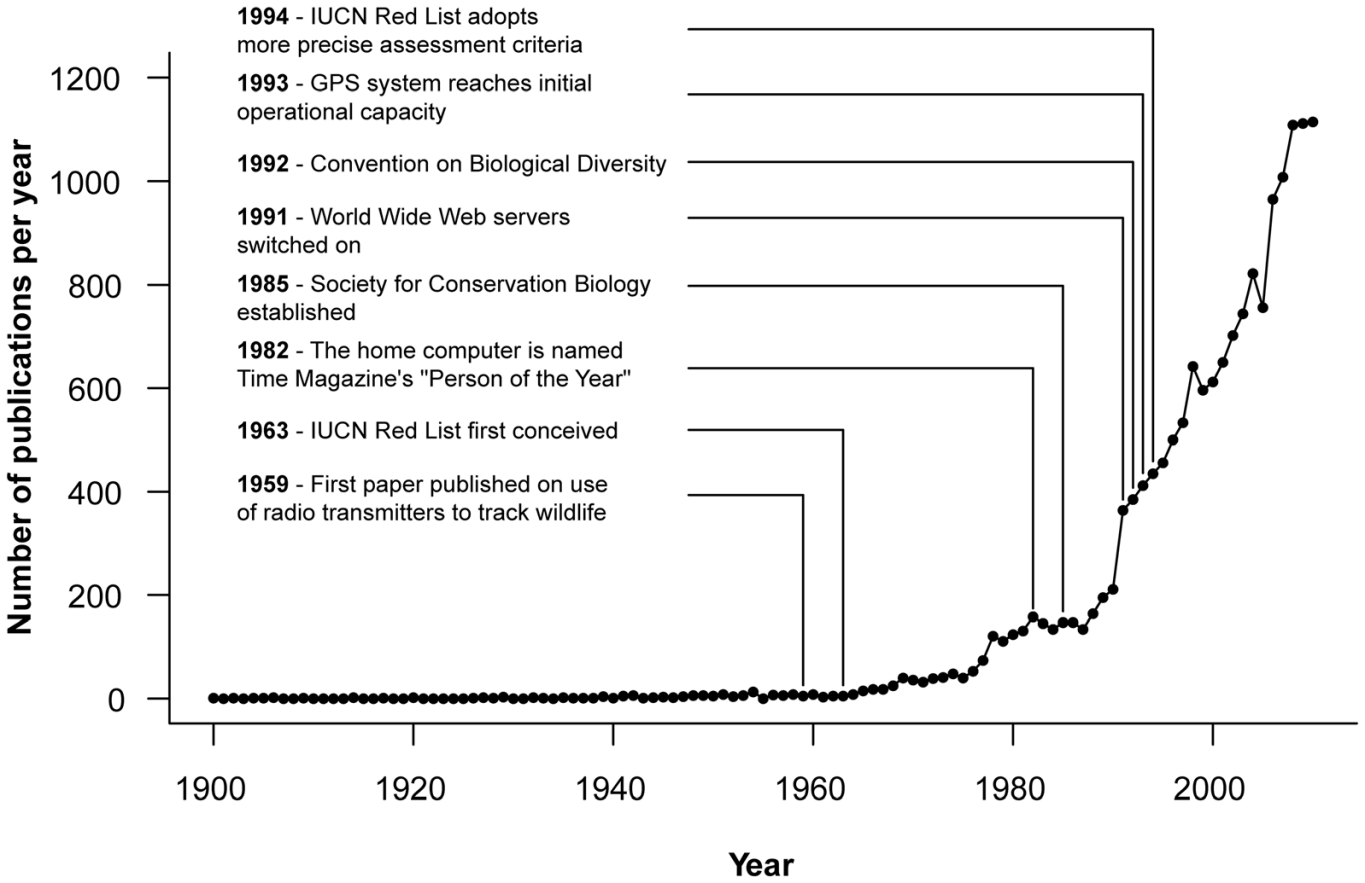
The number of Carnivore papers published per year from 1900–2010. A number of notable dates are also shown.

**Figure 2:**
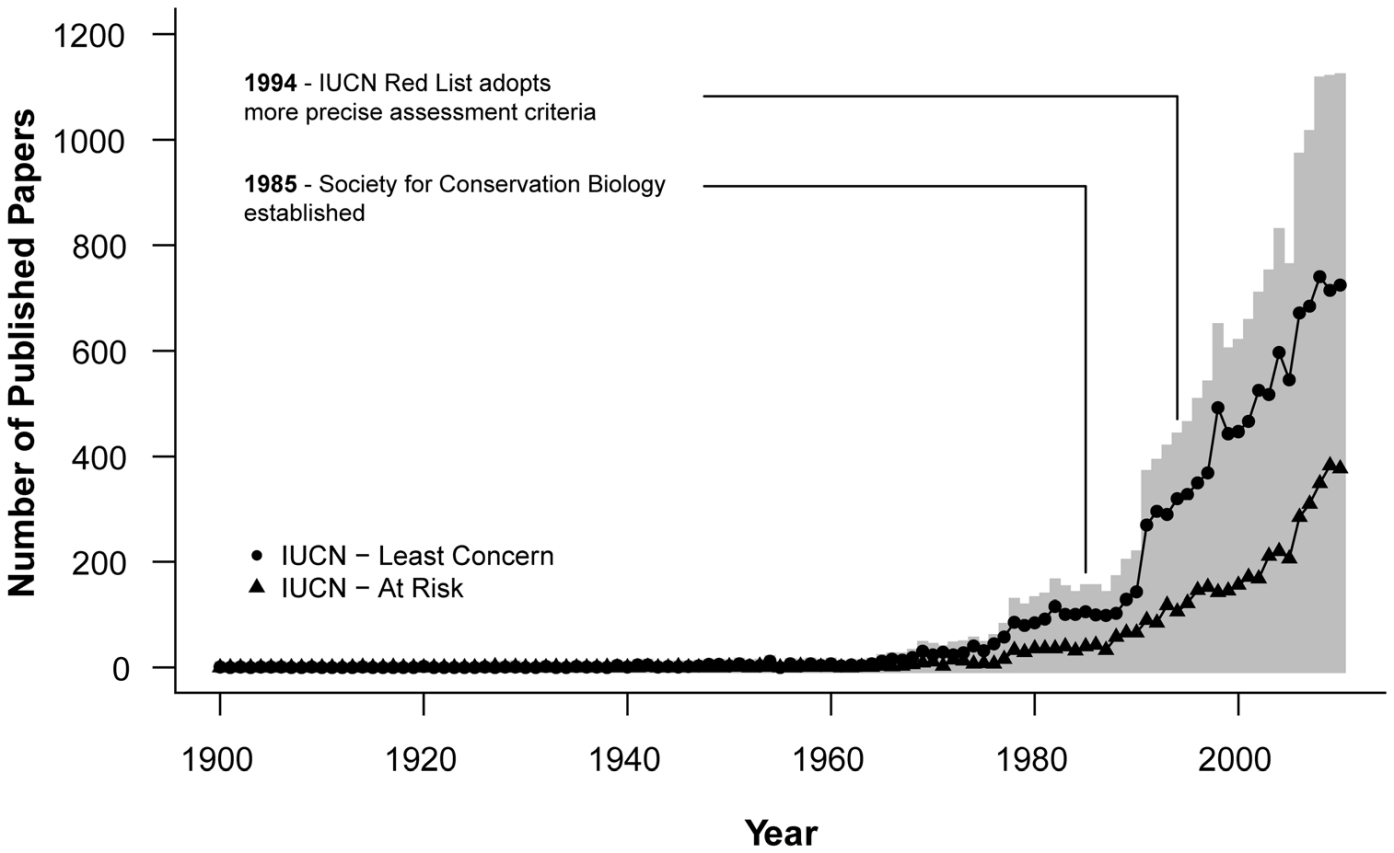
The number of Carnivore papers published per year from 1900–2010 for species of Least Concern and at-risk species (IUCN Near Threatened, Vulnerable, Endangered and Critically Endangered). The barplot shows the total number of papers per year.

The dataset showed an overall increasing trend in the annual publication rate (Figure 1). However, the proportion of papers concerning at-risk species (IUCN Near Threatened, Vulnerable, Endangered and Critically Endangered) did not show a significant change in temporal trend associated with either the introduction of conservation biology as an academic discipline in 1985 or the change in assessment criteria of the IUCN Red List in 1994 (Figure 2, Table S2).

### Most studied Carnivores by Family

In terms of absolute number of papers, Canidae (dogs) were the subject of the most papers (*n* = 3387) and Nandiniidae (African palm civet) the fewest (*n* = 4) (Table 1). We calculated the mean number of papers per species for each Family. Using this method, Nandiniidae remained the least studied Family, Ursidae (bears, *n* = 250) had the most published papers per species and Canidae moved to fourth position (*n* = 97).

**Table 1:** Summary of Carnivore Families ordered by number of papers published per Species.

Family	Mean number of papers per species	Number of species (*n* = 286)	Number of papers (*n* = 16, 367)	Example of species within Family
Ursidae	250.25	8	2002	Bears
Odobenidae	145	1	145	Walrus
Phocidae	101.36	19	1925	“True” seals
Canidae	96.77	35	3387	Dogs
Hyaenidae	79.75	4	319	Hyaenas, Aardwolf
Otariidae	77.88	16	1246	Fur seals, sealions
Felidae	74.20	40	2968	Cats
Ailuridae	53	1	53	Red panda
Mustelidae	49.37	59	2913	Badgers, weasels, otters
Procyonidae	42.79	14	599	Raccoons, coatis
Mephitidae	18.42	12	221	Skunks
Herpestidae	9.91	33	327	Mongooses, meerkat
Viverridae	6.29	35	220	Civets, genets
Eupleridae	4.75	8	38	Madagascan endemics
Nandiniidae	4	1	4	African palm civet

The number of papers published per Family is also shown.

### Least studied Carnivores by Family

Twenty eight Carnivore Species from seven Families had zero published papers (Table 2), including over a quarter of Herpestidae (*n* = 9, 27%) and a fifth of Procyonidae (*n* = 3, 21%). The three least studied Families had similar mean numbers of papers per species although the size of the Family varied from a single species (Nandiniidae) to 35 species (Viverridae). Of these, only the Viverridae had species with zero published papers (*n* = 5, 14%).

**Table 2:** The seven Carnivore Families containing Species with no published papers, showing the percentage of Species with zero published papers within each Family in descending order.

Family	Examples of Species within Family	Number of Species	Number of Species with 0 papers	Percentage of total number of Species in the Family (%)
Herpestidae	Mongooses, meerkat	33	9	27.3
Procyonidae	Raccoons, coati	14	3	21.4
Viverridae	Civets, genets	35	5	14.3
Mustelidae	Badgers, weasels, otters	59	6	10.2
Mephitidae	Skunks	12	1	8.3
Felidae	Cats	40	3	7.5
Canidae	Dogs	35	1	2.9

### Most studied Carnivores

Analysing the data at species level, seven of the 15 Carnivore Families comprised the 20 most published species with Ursidae being proportionally the most represented Family (*n* = 3, 37.5%). The most published species was the red fox (*Vulpes vulpes*, *n* = 923), very closely followed by *Canis lupus* (*n* = 919) which includes 37 sub-species: 35 *C. lupus* wolf sub-species, *C. lupus dingo* (dingo) and *C. lupus familiaris* (domestic dog) [21]. Brown bear was the third most published species (*Ursus actos*, *n* = 787; see Table 3).

**Table 3:** The 20 most studied Carnivore species by number of peer-reviewed published papers.

Top 20 most published Carnivores		*n* papers
Red fox	*Vulpes vulpes*	923
Wolf	*Canis lupus* *	919
Brown bear	*Ursus arctos*	787
Harbour seal	*Phoca vitulina*	580
Eurasian badger	*Meles meles*	547
American black bear	*Ursus americanus*	531
Domestic cat	*Felis catus*	468
Coyote	*Canis latrans*	454
Raccoon	*Procyon lotor*	446
Puma	*Puma concolor*	381
European otter	*Lutra lutra*	369
Lion	*Panthera leo*	318
Grey seal	*Halichoerus grypus*	316
Polar bear	*Ursus maritimus*	299
Southern elephant seal	*Mirounga leonina*	249
Tiger	*Panthera tigris*	247
Spotted hyaena	*Crocutua crocuta*	237
European polecat	*Mustela putorius*	233
Cheetah	*Acinonyx jubatus*	232
Ermine	*Mustela erminea*	228

* We followed Wilson & Reeder's (2005) mammalian taxonomy [29], consequently *C. lupus* includes 37 sub-species: *C. lupus dingo*, *C. lupus familiaris* (domestic dog) and 35 *C. lupus* wolf sub-species.

### Extinction risk and Carnivore research effort 1995–2010

There were 12,201 Carnivore papers published from 1995 to 2010 (inclusive). In this subset, the median number of papers per species was 8 (mean 43; range 0–742). There were minor changes in position within the top 20 most studied species 1995–2010 when compared with the top 20 most studied 1901–2010 (Table S3). There were only two species which were no longer included (European polecat, *Mustela putorius* and ermine, *Mustela erminea*). Due to tied numbers of papers at positions 14 and 20, three species replaced them (European lynx, *Lynx lynx*; Steller sealion, *Eumetopias jubatus* and giant panda, *Ailuropoda melanoleuca*).

Among the 20 most published Carnivores 1995–2010, the majority were globally non-threatened (*n* = 13, 62%). Of the remaining species, three were Endangered (tiger, *Panthera tigris*; Steller sealion *Eumetopias jubatus*; giant panda *Ailuropoda melanoleuca*), three were Vulnerable (lion, *Panthera leo*; polar bear, *Ursus maritimus*; cheetah, *Acinonyx jubatus*), one was Near Threatened (European otter, *Lutra lutra*) and the domestic cat (*Felis catus*) was not listed by the IUCN. The results of our Kruskal-Wallis testing allowed us to reject the null hypotheses that IUCN categories had the same mean rank and therefore the same level of research effort (H = 28.3381, 7 d.f., *p*<0.001).

Species classified as being at a high risk of extinction had the highest median number of papers (Critically Endangered *n* = 26 and Endangered *n* = 17.5), followed by those of Least Concern (*n* = 10; see Table 4). There were seven Critically Endangered Carnivores [33]; when all species were ranked by number of papers, the first Critically Endangered species was ranked 52^nd^ (Iberian lynx, *Lynx pardinus n* = 57). One Critically Endangered species, the Malabar large-spotted civet (*Viverra civettina*), had zero published papers.

**Table 4:** Research effort by IUCN Red List status showing the mean and median number of papers per species published 1995–2010 for each IUCN threat category.

IUCN Red List Status	Number of species (*n* = 286)	Number of papers (*n* = 12,201)	Mean number of papers per species	Median number of papers per species
Critically endangered	7	177	25	26
Endangered	24	1090	45	17.5
Vulnerable	37	1205	33	5
Near threatened	27	694	26	7
Least concern	163	8538	52	10
Data deficient	19	156	8	0
Not IUCN listed*	5	335	67	2
Extinct	4	6	1.5	1

* 5 unlisted felines: domestic cat (*Felis catus*), Chinese mountain cat (*F. bieti*), Pantanal cat (*Leopardus braccatus*), Pampas cat (*L. pajeros*), Iriomote cat (*Prionailurus iriomotensis*).

The majority of the 19 species classified by the IUCN as Data Deficient had two or fewer published papers (79%, *n* = 15). *Post-hoc* pair-wise Wilcoxon tests with Bonferroni corrections revealed that Data Deficient species had significantly fewer published papers than Vulnerable (*p*<0.05), Least Concern and Endangered species (both *p*<0.001). These Data Deficient species also accounted for a quarter of those Carnivores with zero published papers.

### Marine Carnivores

There were 3476 papers on the 37 species of marine Carnivore, accounting for 21% of all Carnivore publications (see Table 5). Univariate GLM for marine Carnivore research effort showed no significant relationship with adult body mass, diet or IUCN status (Table S4)

**Table 5:** Summary of marine Carnivore research effort showing the mean and median number of papers per species.

Species		Mean number of papers per species	Median number of papers (Range)	Number of species (*n* = 286)	Number of papers (*n* = 16, 367)
All terrestrial		51.77	10 (0–923)	249	12, 891
All marine		93.95	60 (1–580)	37	3476
Sea otter	*Enhydra lutris*	160	NA	1	160
Family Odobenidae	*Odobenus rosmarus*	145	NA	1	145
Family Phocidae	Seals	101.36	58 (6–580)	19	1925
Family Otariidae	Eared seals	77.88	55.5 (1–206)	16	1246

The number of papers published for terrestrial Carnivores is shown for comparison.

### Characteristics associated with terrestrial Carnivore research effort

Multivariate GLM model simplification is shown in Table S5. No interactions were found to be significant. Exclusion of Family was not supported by model selection testing, suggesting a suppression effect of Family. The minimal adequate model is shown in Table 6.

**Table 6:** Minimal adequate GLM for research effort.

	Estimate	Std. Error	z value	Standardized Beta coefficient
Intercept	1.3521	0.8752	1.545	
log10 Adult body mass (g)	0.8931	0.2065	4.325***	4.7261e-03
√Range km^2^	0.0003	0.0001	3.927***	3.1812e-03
Diet	−0.2541	0.0516	−4.928***	−2.9784
Family Ailuridae	1.4620	1.6290	0.897	
Family Canidae	1.3480	1.3380	1.007	
Family Eupleridae	0.1452	1.2940	0.112	
Family Felidae	0.3431	1.3880	0.247	
Family Herpestidae	0.7554	1.2490	0.605	
Family Hyaenidae	−0.0309	1.510	−0.020	
Family Mephitidae	1.9170	1.3540	1.416	
Family Mustelidae	1.2670	1.2930	0.980	
Family Nandiinidae	−1.907	1.7160	−1.111	
Family Procyonidae	1.9100	1.3530	1.411	
Family Ursidae	1.7230	1.6540	1.042	
Family Viverridae	−0.2730	1.4020	−0.195	

***p<0.001.

Research effort is measured as a count of the number of published papers using negative binomial distribution and log-link. Null deviance 265.47 (105 d.f.); Residual deviance 118.89 (91 d.f.).

## Discussion

In our study of almost 16,500 peer-reviewed publications we have quantified research effort across the entire Order Carnivora and have reported considerable disparity at both Family and Species level whilst the number of published papers has increased annually since the late 1960s (Figure 1). The annual increase in the number of scientific articles and the journals which publish them is well documented [43]–[45] and is likely to be due to numerous factors, including changes in academic publishing and methods of communication which are difficult to quantify. Similarly, advances in telemetry and associated technologies have enabled detailed studies, particularly of elusive species and those in hard-to-reach habitats, but again it was not possible to capture this in a single variable.

Ecological and life history traits associated with being well-studied were larger body size and larger geographic range size whilst diet was a negative predictor meaning that species with broader diets (omnivores) were generally less studied. Although we identified a number of differences between Families and between IUCN extinction risk categories, neither were found to be good predictors of research effort although Family exerted some suppression effect on our minimal adequate GLM. Neither habitat breadth nor activity cycle, both potential indicators of species accessibility to researchers, were significant predictors of research effort. Marine Carnivores were well-studied relative to their terrestrial counterparts although neither extinction risk, adult body mass nor diet were found to be significant predictors of research effort, suggesting that other factors drive marine Carnivore research.

### A reflection of human perspectives?

Larger, more widely distributed Carnivores have tended to be described earlier [46] which could contribute to the greater number of papers being published on these species. Further, species with larger geographic ranges tend to be larger bodied and move further increasing the chance of interaction with humans [46]. It is well documented that scientists are more likely to work in accessible locations such as established protected areas, locations which are close to research institutions (or their outposts) or more highly human populated areas and are much less likely to conduct research in countries which lack infrastructure or which are politically unstable [10], [15], [18]–[21]. Our findings may reflect the role of human perspectives in research, for example a number of the particularly well-studied species may be perceived as a “nuisance” either posing a disease threat or coming into conflict with humans over shared resources.

Carnivore species of all sizes may come into conflict with humans over resources such as food or space [47], [48] and large Carnivores may attack humans [49]. The majority of the most studied Carnivores (Tables 3 and S3) are in conflict with humans to some degree whether as direct threat, by competing for resources or as vectors for disease transmission. The need to manage the human-Carnivore relationship is not new [47], [48] although the frequency and intensity of human-Carnivore conflict may be rising as human populations expand with concurrent loss of Carnivore habitat (e.g. tigers *Panthera tigris* and leopards *P. pardus* in India). In other areas, conflict may arise due to increasing Carnivore populations following successful recovery and management programs (e.g. predation of livestock by bears *Ursus spp*. and wolves *Canis lupus* in Europe).

There was a negative relationship between HPD and research effort, although it was not a significant factor in the multivariate model. These less-studied species were mainly endemic to Asia, the most densely human-populated continent on Earth [50] and specifically from Central, Eastern and South-East Asia. The high levels of human density and rapid development in many of these countries could account for the apparent lack of Carnivore research – there may not be the skills or resources available and/or there may be more pressing priorities.

### Species-level predictors of research effort

Our finding that Family itself did not predict research effort suggests that Species-level traits and characteristics (such as body mass) are more important predictors of research attention than taxonomic group *per se*. It has previously been suggested that some species are simply more difficult to study than others which significantly contributes to disparity in research effort [51] and it is not unlikely that some of our findings reflect similar issues among Carnivores. Ecological characteristics which may contribute to difficulty in studying these species include being solitary, nocturnal, arboreal, some inhabit difficult terrain such as deserts, mountainous or politically unstable regions whilst many are fairly small (e.g. 94% of the Carnivores with zero published papers had known adult body mass and all but one weighed ≤2.4 kg). Taxonomic uncertainty may have contributed to the apparent lack of research effort on some species, as is the case for ten of the 28 Carnivores with zero published papers. For example, there is considerable discussion as to whether the three species of olingo (*Bassaricyon beddardi*, *B.lasius* and *B.pauli*) are sub-species rather than three separate species [29].

Diet was a significant negative predictor of research effort in our multivariate model, meaning that species with broader diets (omnivores) were less studied. In addition to their broad diets, these omnivores are also highly adaptable in terms of habitat use and are often common i.e. widespread and abundant. Whilst they are often associated with humans and thus potentially more convenient to study, these adaptable generalists would not be the obvious choice for studying popular ecological topics such as predator/prey dynamics or foraging. There were however, four particularly well-studied omnivores with over 100 papers each: brown bear (*U. arctos*); striped skunk (*Mephitis mephitis*); racoon dog (*Nyctereutes procyonoides*) and racoon (*Procyon lotor*). These well-studied omnivores are all widespread, abundant and, possibly most significantly, can be a nuisance to humans including acting as vectors of disease that affect both humans and domestic animals.

**Figure 3:**
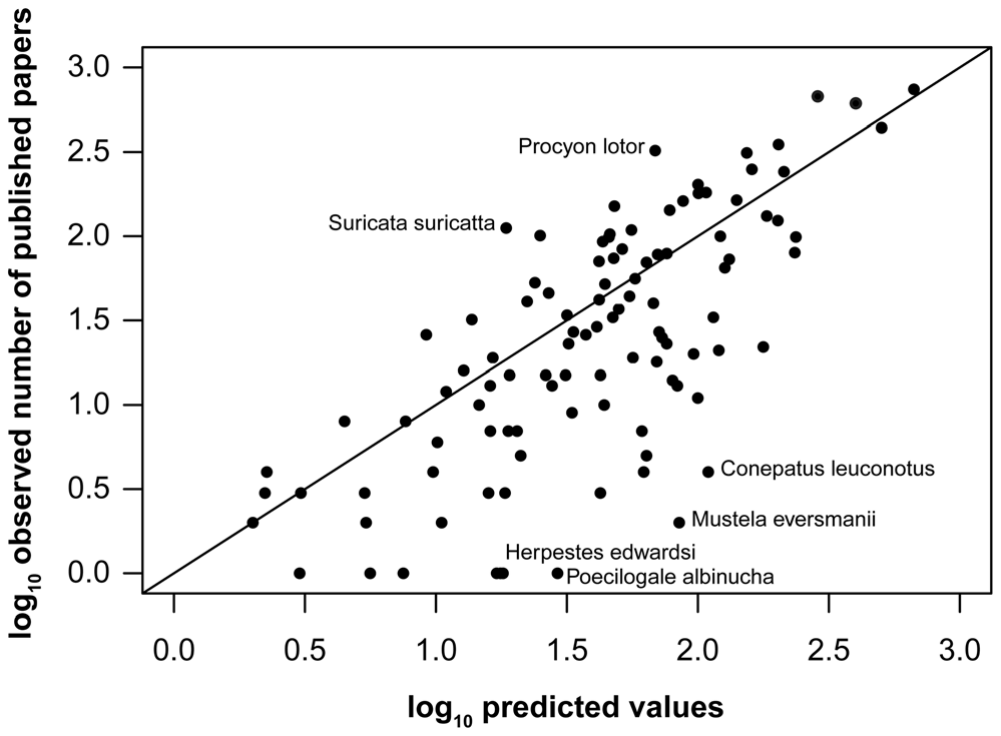
Analysis of model residuals: Predicted Vs Observed values. The line indicates where the predicted values  =  observed values i.e. the model is a perfect fit to the data. Species which receive more research attention than our model predicts based on their biology are above the line whilst those receiving less are below the line. Species with residuals greater than +2 or −2 are labelled.

Analysis of our model residuals (Figure 3) identified two species which received much greater study effort than their biology would predict, meerkat (*Suricata suricatta*) and raccoon (*P. lotor*). The complex meerkat social system is well-studied by social and behavioural ecologists whilst raccoons are very widespread, often in close contact with humans. Four species received much less research effort than our model would predict based on their biology: the “secretive” [33] African striped weasel (*Poecilogale albinucha*), the abundant and widespread Indian grey mongoose (*Herpestes edwardsi*), Steppe polecat (*Mustela eversmanii*, widely considered conspecific with the European polecat) and the nocturnal, solitary American hog-nosed skunk (*Conepatus leuconotus*).

The only other study to explore Carnivore research effort in a similar manner focused exclusively on the Felidae [17]. There was a high level of agreement between the two studies when we compared the ten most published cat species. When the domestic cat was excluded from our dataset, whilst some of the rankings differed, the top ten most published cat species were almost identical. The wild cat (*Felis silvestris*) was the only species to appear in our top ten but not the earlier study where it was ranked 15^th^ by number of published papers. In the Felidae study body size was found to be the only significant predictor of research effort, with larger cats being studied more intensively [17] for which the author offered three possible explanations: larger cats potentially interact more with humans, are easier to observe and are more charismatic thus attracting researchers to study them. We found that body size was a predictor of research effort across all terrestrial Carnivore Families. While it is difficult to identify which factors are motivating researchers' interest in these species, future research on the content of these papers may be used to begin to do this.

### Carnivore extinction risk and research effort

It has been demonstrated that Carnivores' extinction risks are partly determined by their biological traits [32], [52], specifically by the interaction between these traits and human population density [32]. In addition to varying levels of extinction risk [33], Carnivores have been shown to respond differently to human threat processes and, as human populations continue to grow, species' traits will become increasingly critical determinants of extinction risk [32]. Although larger bodied mammals generally have higher extinction risk [53], previous studies have found that extinction risk is not a driver of research effort [2], [4], [5]. Direct comparisons with our findings are difficult due to methodological differences: two studies were conducted at higher taxonomic scales [4], [5], one was based on publications in four conservation journals [2] and the Felidae study used only in-situ conservation papers [17].

Whilst we were able to clearly demonstrate a year-on-year increase in the Carnivore literature, we were unable to demonstrate any increase in the intensity of study of at-risk species associated with either the establishment of conservation biology as a recognised discipline (widely accepted to have occurred at the Second International Conference on Conservation Biology in 1985) or the adoption of more rigorous IUCN assessment criteria in 1994. Extinction risk was not a significant predictor of research effort in our GLM but two different patterns emerged from summaries of the data depending on the measure of effort used. Using the mean number of papers per species suggests that those at lower risk of extinction are more intensively studied, a similar finding to earlier studies [2]–[5] whilst using the median number of papers to account for extreme values suggests that the most vulnerable species (Endangered and Critically Endangered) are the most studied.

Despite this general finding, our study has also revealed areas of disparity between research effort and extinction risk, highlighting potential priority areas for future work. The most striking example are the Madagascan endemic Eupleridae which are one of the most threatened Carnivore Families (one Endangered, three Vulnerable, three Near Threatened and one Least Concern) but were the second least studied Family by total publications (*n* = 38), mean number of papers per species (*n* = 5) and the third least studied based on median number of papers per species (*n* = 3). The combination of intrinsic vulnerability due to life history traits and extrinsic environmental and spatial pressures are known to increase species' vulnerability to decline [32], [54]–[56]: the Eupleridae typically have restricted range size and low population densities which are exacerbated by numerous extrinsic threats including human hunting, predation by non-native Carnivores, habitat loss caused by landscape changes [33] and Madagascar's sustained high human population growth rate [57]. Cardillo *et al.* [32] argue that African viverrid species may be particularly vulnerable to threats, requiring pre-emptive action to maintain their current non-threatened status. It is clear from our study that more research effort on these species is needed for us to develop effective conservation action for this group.

The work which we have reported here asks which species are most commonly studied but future exploration of how research topics vary across species may provide better understanding of our motivations for studying them. Further bibliometric analyses of our dataset will enable us to explore underlying patterns in the Carnivore literature to begin to understand what it is about particular species and/or traits that means that they are particularly studied.

## Conclusions

We are the first, to our knowledge, to combine bibliometric information with global datasets on the ecology and life history traits of an entire Order. Our extensive study of 286 Carnivores included almost 16, 500 papers published in 534 journals between 1900–2011. We have identified that there is a wide variation in intensity of research effort at both Family and Species levels, with some of the least studied being those which may need protection in future. Rather than being driven by other characteristics including extinction risk, Carnivore research appears to be driven by their body size, range size and to some extent diet. Some of our findings hint at the complex role of human perspectives and the need to manage human-Carnivore relationships in setting research agendas. Our findings could be combined with other resources to prioritise and co-ordinate future research effort and resources.

More generally, our work demonstrates another combination of literature-search- based bibliometric analysis with specialist knowledge to quantify and interpret knowledge gap findings. This growing suite of relatively easy-to-use methods to describe knowledge gaps can be applied across many scientific disciplines.

## Supporting Information

Figure S1 **Percentage coverage of each life history variable.** All data from PanTHERIA (Jones *et al*. 2009) except extinction risk (“IUCN”) taken from the IUCN Red List (IUCN 2011). The first panel shows all 286 Carnivores, subsequent panels show the percentage coverage of each variable by Family. Note that Ailuridae, Eupleridae, Hyaenidae and Nandiinidae are not shown as there were no missing data.
(PDF)

Table S1 **A. Generalised Variance Inflation Factor (GVIF) for full multivariate GLM.** NB GVIF rather than VIF is provided as at least one term has >1 d.f. GVIF ^1/2d.f.^ is also shown. **B. Generalised Variance Inflation Factor (GVIF) for minimal adequate GLM.** NB GVIF rather than VIF is provided as at least one term has >1 d.f. GVIF ^1/2d.f.^ is also shown.
(DOCX)

Table S2 **Proportional time series binomial GLM.**
(DOCX)

Table S3 **The 20 most studied Carnivore species by number of peer-reviewed published papers 1995–2010.** Note tie at positions 14 (*n* = 203) and 20 (*n* = 162).
(DOCX)

Table S4 **Univariate negative binomial GLM to analyse the individual effects of body mass, IUCN extinction risk and diet on research effort on marine Carnivores.** Research effort is measured as a count of the number of published papers using negative binomial distribution and log-link. There are 37 marine Carnivores, a total of 34 were used in the GLM: Of the three excluded, two are extinct (*Zalophus japonicus* and *Monachus tropicalis*) and there was no body mass available for *Zalophus wollebaeki*.
(DOCX)

Table S5 **Summary of GLM simplification.**
(DOCX)

Material S1 **Web of Science Categories (Based on Science Citation Index & SCI-Expanded).** Journals may appear in more than 1 category.
(DOCX)
