# Supplementary figures and images for: Correlates of Research Effort in Carnivores: Body Size, Range Size and Diet Matter
Source: PLoS One. 2014 Apr 2;9(4):e93195. doi: 10.1371/journal.pone.0093195 (PMC3973602; doi:10.1371/journal.pone.0093195)

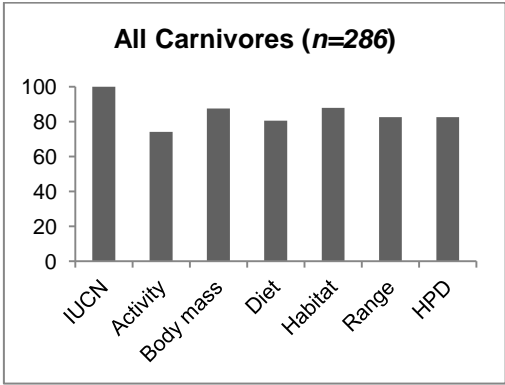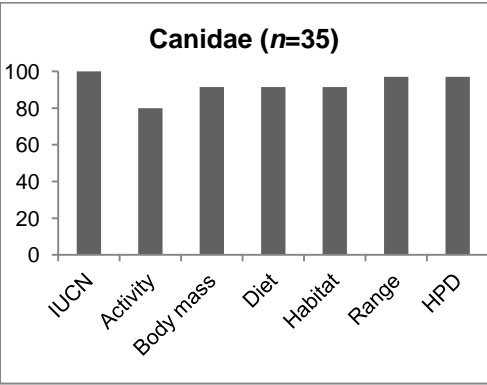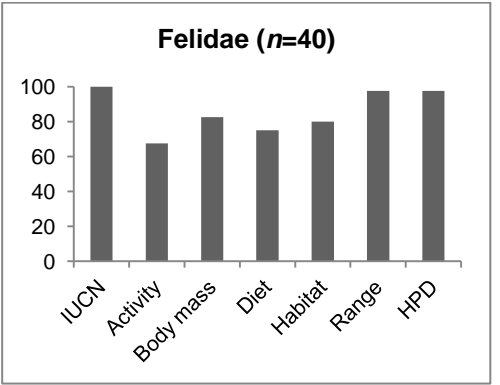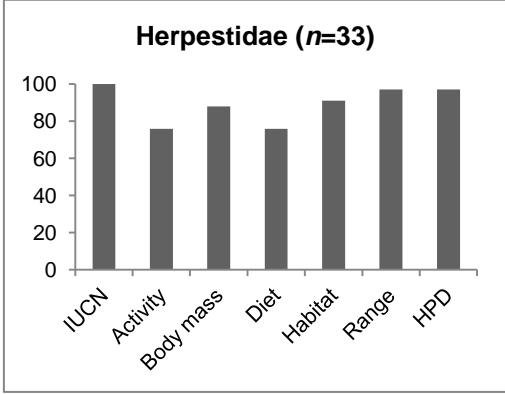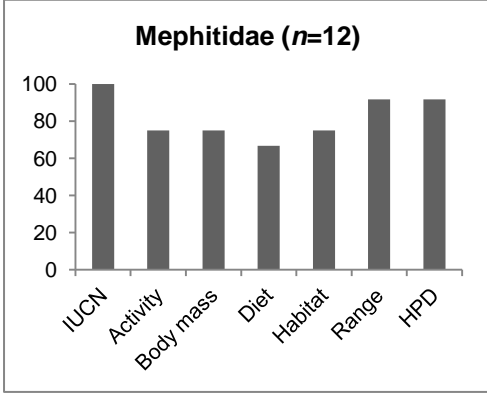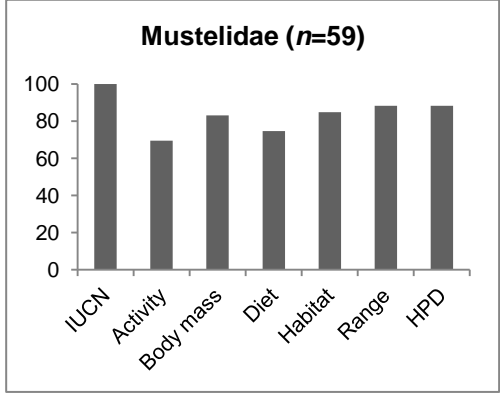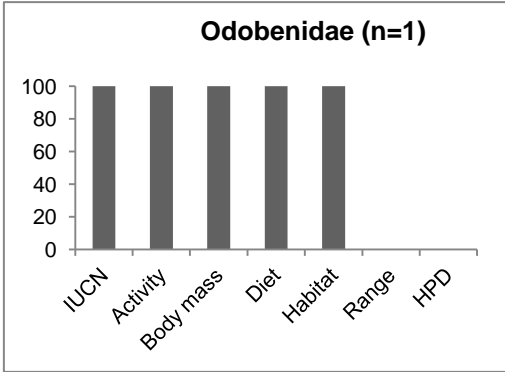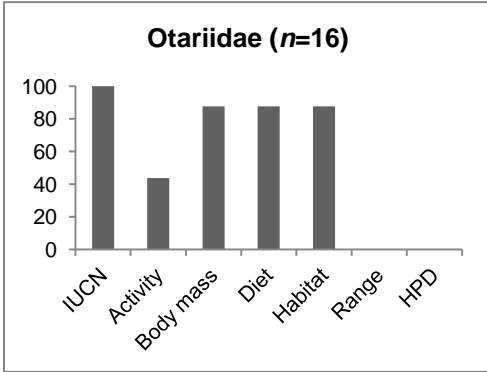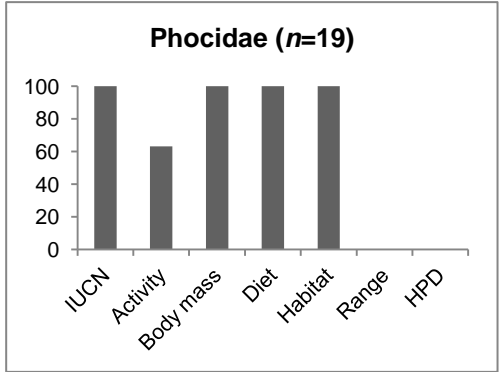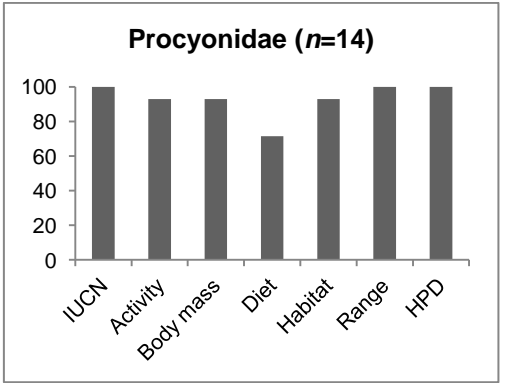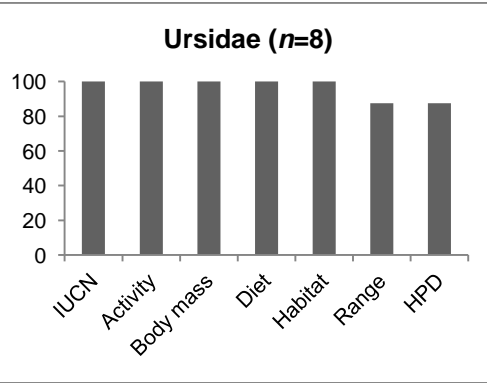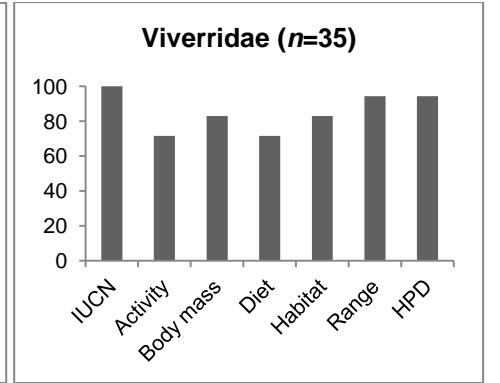

Supplement: Figure S1 — Percentage coverage of each life history variable. All data from PanTHERIA (Jones et al. 2009) except extinction risk (“IUCN”) taken from the IUCN Red List (IUCN 2011). The first panel shows all 286 Carnivores, subsequent panels show the percentage coverage of each variable by Family. Note that Ailuridae, Eupleridae, Hyaenidae and Nandiinidae are not shown as there were no missing data. (PDF) [file pone.0093195.s001.pdf]
