# Supplementary material for: Correlates of Research Effort in Carnivores: Body Size, Range Size and Diet Matter
Source: PLoS One. 2014 Apr 2;9(4):e93195. doi: 10.1371/journal.pone.0093195 (PMC3973602; doi:10.1371/journal.pone.0093195)
Supplement: Table S1 — A. Generalised Variance Inflation Factor (GVIF) for full multivariate GLM. NB GVIF rather than VIF is provided as at least one term has >1 d.f. GVIF 1/2d.f. is also shown. B. Generalised Variance Inflation Factor (GVIF) for minimal adequate GLM. NB GVIF rather than VIF is provided as at least one term has >1 d.f. GVIF 1/2d.f. is also shown. (DOCX) [file pone.0093195.s002.docx]

Table S1A.

|  | **GVIF** | **d.f.** | **GVIF ^1/2d.f.^** |
| --- | --- | --- | --- |
| log_10_ Adult body mass | 2.5258 | 1 | 1.5893 |
| √Geographic range area | 1.4496 | 1 | 1.2040 |
| log_10_ Mean human density | 1.1810 | 1 | 1.0867 |
| IUCN | 1.6462 | 1 | 1.2831 |
| Habitat | 2.9733 | 3 | 1.1991 |
| Diet | 1.7221 | 1 | 1.3123 |
| Family | 12.0862 | 11 | 1.1199 |

Table S1B.

|  | **GVIF** | **d.f.** | **GVIF ^1/2d.f.^** |
| --- | --- | --- | --- |
| log_10_ Adult body mass | 2.3098 | 1 | 1.5198 |
| √Geographic range area | 1.2471 | 1 | 1.1167 |
| Diet | 1.6308 | 11 | 1.2770 |
| Family | 4.3410 | 1 | 1.0690 |
