# Supplementary material for: Correlates of Research Effort in Carnivores: Body Size, Range Size and Diet Matter
Source: PLoS One. 2014 Apr 2;9(4):e93195. doi: 10.1371/journal.pone.0093195 (PMC3973602; doi:10.1371/journal.pone.0093195)
Supplement: Table S2 — Proportional time series binomial GLM. (DOCX) [file pone.0093195.s003.docx]

|  | **Estimate** | **Std. Error*** | **z value** | ***p* value** |
| --- | --- | --- | --- | --- |
| Intercept | 1.1140 | 0.6520 | 17.0700 | < 0.001 |
| Conservation Biology Introduction (1985) | -0.1720 | 0.1170 | -1.4690 | 0.1420 |
| IUCN Red List assessment criteria updated (1994) | -0.0460 | 0.2380 | -0.1930 | 0.8470 |

* Newey-West Standard Errors

Residual deviance 128.15 (48 d.f.)
