# Supplementary material for: Correlates of Research Effort in Carnivores: Body Size, Range Size and Diet Matter
Source: PLoS One. 2014 Apr 2;9(4):e93195. doi: 10.1371/journal.pone.0093195 (PMC3973602; doi:10.1371/journal.pone.0093195)
Supplement: Table S4 — Univariate negative binomial GLM to analyse the individual effects of body mass, IUCN extinction risk and diet on research effort on marine Carnivores. Research effort is measured as a count of the number of published papers using negative binomial distribution and log-link. There are 37 marine Carnivores, a total of 34 were used in the GLM: Of the three excluded, two are extinct (Zalophus japonicus and Monachus tropicalis) and there was no body mass available for Zalophus wollebaeki. (DOCX) [file pone.0093195.s005.docx]

|  | **Estimate** | **Std. Error** | **Deviance** | **d.f.** | **z value** |
| --- | --- | --- | --- | --- | --- |
| Intercept | 4.4650 | 0.2042 | 39.8090 | 33 | 21.8680 |
| Adult body mass | 5.1440 | 4.828e-07 | 38.3440 | 32 | 1.0660 |
| Intercept | 4.9240 | 0.2663 | 40.6250 | 33 | 18.4910 |
| IUCN | -0.1426 | 0.0911 | 38.2550 | 32 | 0.1170 |
| Intercept | 5.2044 | 0.3674 | 41.8590 | 33 | 14.1670 |
| Diet | -0.1359 | 0.0716 | 38.1370 | 32 | -1.8970 |
