# Supplementary material for: Correlates of Research Effort in Carnivores: Body Size, Range Size and Diet Matter
Source: PLoS One. 2014 Apr 2;9(4):e93195. doi: 10.1371/journal.pone.0093195 (PMC3973602; doi:10.1371/journal.pone.0093195)
Supplement: Table S5 — Summary of GLM simplification. (DOCX) [file pone.0093195.s006.docx]

| **Model** | **Term dropped** | **AIC** | **Model d.f.** | **LR test *p* value** |
| --- | --- | --- | --- | --- |
| Model 1 |  | 1036.2 | 86 |  |
| Model 1 + Mass*Range*HPD |  | 1045.1 | 82 | 1.000 (vs. model 1) |
| Model 1 + Mass*Range | *HPD | 1038.2 | 85 | 0.7882 (vs. model 1) |
| Model 1 + Mass*Range*IUCN |  | 1039.7 | 81 | 0. 2545 (vs. model 1) |
| Model 1 + Mass*IUCN | *Range | 1038.2 | 85 | 0. 9422 (vs. model 1) |
| Model 2 | Mass*IUCN, IUCN | 1034.3 | 87 | 0.8483 (vs. model 1) |
| Model 3 | Habitat | 1031.2 | 90 | 0.4117 (vs. model 2) |
| Model 3 + Mass*Range*HPD |  | 1036.8 | 86 | 0.6790 (vs. model 3) |
| Model 3 + Mass*Range | *HPD | 1033 | 89 | 0.6793 (vs. model 3) |
| Model 4 | Mass*Range, HPD | 1029.7 | 91 | 0.4465 (vs. model 3) |
| Model 4 + Mass*Range |  | 1031.4 | 90 | 0.5665 (vs. model 4) |
| Model 5 | Mass*Range, Family | 1039.9 | 102 | 0.0007 (vs. model 4) |

**Model 1** *n* papers ~ Mass + Range + HPD + IUCN + Habitat + Diet + Family

**Model 2** *n* papers ~ Mass + Range + HPD + Habitat + Diet + Family

**Model 3** *n* papers ~ Mass + Range + HPD + Diet + Family

**Minimal adequate model: Model** **4** *n* papers ~ Mass + Range + Diet + Family

**Model 5** *n* papers ~ Mass + Range + Diet
