# Supplementary material for: Correlates of Research Effort in Carnivores: Body Size, Range Size and Diet Matter
Source: PLoS One. 2014 Apr 2;9(4):e93195. doi: 10.1371/journal.pone.0093195 (PMC3973602; doi:10.1371/journal.pone.0093195)
Supplement: Material S1 — Web of Science Categories (Based on Science Citation Index & SCI-Expanded). Journals may appear in more than 1 category. (DOCX) [file pone.0093195.s007.docx]

**Anatomy & Morphology**
**Behavioural Sciences**
**Biodiversity Conservation**
**Biology**
**Developmental Biology**
**Ecology**

**Environmental Studies**

**Environmental Science**
**Evolutionary Biology**

**Forestry**

**Genetics & Heredity**
Marine & Freshwater Biology
Multidisciplinary Sciences Includes “Science” & “Nature”.
**Reproductive Biology**
**Veterinary Sciences**
**Zoology**
