# Supplementary material for: Correlates of Research Effort in Carnivores: Body Size, Range Size and Diet Matter
Source: PLoS One. 2014 Apr 2;9(4):e93195. doi: 10.1371/journal.pone.0093195 (PMC3973602; doi:10.1371/journal.pone.0093195)
Supplement: Table S3 — The 20 most studied Carnivore species by number of peer-reviewed published papers 1995–2010. Note tie at positions 14 (n = 203) and 20 (n = 162). (DOCX) [file pone.0093195.s004.docx]

| **Top 20 most published carnivores 1995-2010** | | ***n* papers** | **Change in rank versus papers published all years** |
| --- | --- | --- | --- |
| Wolf | *Canis lupus** | 742 | +1 |
| Brown bear | *Ursus arctos* | 674 | +1 |
| Red fox | *Vulpes vulpes* | 613 | -2 |
| American black bear | *Ursus americanus* | 440 | +2 |
| Harbour seal | *Phoca vitulina* | 383 | -1 |
| Eurasian badger | *Meles meles* | 361 | -1 |
| Coyote | *Canis latrans* | 351 | +1 |
| Domestic cat | *Felis catus* | 328 | -1 |
| Raccoon | *Procyon lotor* | 323 | = |
| Puma | *Puma concolor* | 313 | = |
| Lion | *Panthera leo* | 250 | +1 |
| Polar bear | *Ursus maritimus* | 245 | +2 |
| European otter | *Lutra lutra* | 242 | -2 |
| Grey seal | *Halichoerus grypus* | 203 | -1 |
| Tiger | *Panthera tigris* | 203 | +2 |
| European lynx | *Lynx lynx* | 182 | +5 |
| Cheetah | *Acinonyx jubatus* | 180 | +2 |
| Steller sealion | *Eumetopias jubatus* | 175 | +6 |
| Giant panda | *Ailuropoda melanoleuca* | 164 | +4 |
| Southern elephant seal | *Mirounga leonina* | 162 | -3 |
| Spotted hyaena | *Crocutua crocuta* | 162 | -5 |
